# UNDERSTANDING ACCEPTANCE OF HEALTHCARE TECHNOLOGY BY OLDER ADULTS: IMPLICATIONS FOR ADOPTION

**DOI:** 10.1093/geroni/igz038.3382

**Published:** 2019-11-08

**Authors:** Maurita T Harris, Wendy A Rogers

**Affiliations:** University of Illinois at Urbana Champaign, Champaign, Illinois, United States

## Abstract

Healthcare Technology (HCT) can support older adults as it is not uncommon for them to be managing one or more chronic diseases at a time. Thus, understanding older adults’ willingness to use HCTs can guide introduction of new technologies to help with their health management. The purpose of the research is to understand what influential factors emerged when older adults considered using new HCTs and how well current models of technology acceptance represented these factors. Twenty-three older adults (age 65-84) with hypertension completed a semi-structured interview to gain insight into these factors. During the interview, participants were first presented with a scenario to imagine and one of three HCTs (blood pressure monitor, electronic pillbox, and multifunctional healthcare robot) to consider. The qualitative coding identified: (a) facilitators: perceived advantages, easy to use, familiar, useful, and advice acceptance from a healthcare provider; (b) barriers: good for others, not good for me, disadvantages, and unfamiliar; and (c) transition factors that can lead to acceptance: with advice acceptance from a healthcare provider. These findings provide recommendations which can inform dissemination of new HCTs. Recommendations include: highlight the facilitators when introducing new HCT, understand the barriers and transition factors to give support where needed, and include the care network (i.e., people knowledgeable about the HTC and health conditions) to recommend the technology.

